# Combined probiotics with vitamin D_3_ supplementation improved aerobic performance and gut microbiome composition in mixed martial arts athletes

**DOI:** 10.3389/fnut.2023.1256226

**Published:** 2023-10-11

**Authors:** Katarzyna Przewłócka, Marcin Folwarski, Mariusz Kaczmarczyk, Karolina Skonieczna-Żydecka, Joanna Palma, Zofia Kinga Bytowska, Sylwester Kujach, Jan Jacek Kaczor

**Affiliations:** ^1^Department of Bioenergetics and Exercise Physiology, Medical University of Gdańsk, Gdańsk, Poland; ^2^Department of Clinical Nutrition and Dietetics, Medical University of Gdańsk, Gdańsk, Poland; ^3^Department of Clinical and Molecular Biochemistry, Pomeranian Medical University in Szczecin, Szczecin, Poland; ^4^Department of Biochemical Research, Pomeranian Medical University in Szczecin, Szczecin, Poland; ^5^Department of Physiology, Gdansk University of Physical Education and Sport, Gdańsk, Poland; ^6^Department of Neurophysiology, Neuropsychology and Neuroinformatics, Medical University of Gdańsk, Gdańsk, Poland; ^7^Department of Animal and Human Physiology, University of Gdańsk, Gdańsk, Poland

**Keywords:** probiotics, gut microbiome, intestinal permeability, MMA athletes, aerobic capacity, inflammation

## Abstract

**Introduction:**

Mixed Martial Arts (MMA) is characterized as an interval sport in which the training program focuses on enhancing both aerobic and anaerobic capacities. Therefore, strategies targeting the intestinal microbiome may be beneficial for MMA athletes. Moreover, vitamin D supplementation may amplify the positive effects of certain bacterial strains. We previously demonstrated that the combined of probiotics and vitamin D_3_ supplementation improved the lactate utilization ratio, total work, and average power achieved during anaerobic tests in MMA. Therefore, this study aimed to investigate whether combined probiotic and vitamin D_3_ ingestion can modify the composition of the gut microbiome and epithelial cell permeability, influence the inflammatory response, and ultimately enhance aerobic capacity.

**Methods:**

A 4-week clinical trial was conducted with 23 male MMA athletes randomly assigned to either the probiotic + vitamin D_3_ (PRO + VIT D) group or the vitamin D_3_ group (VIT D). The trial employed a double-blind, placebo-controlled design and involved measurements of serum inflammatory markers, gut microbiome composition, epithelial cell permeability, and aerobic performance.

**Results:**

After 4-week of supplementation, we found a significantly lower concentration of calprotectin in the PRO + VIT D group (34.79 ± 24.38 mmol/L) compared to the value before (69.50 ± 46.91) supplementation (*p* = 0.030), augmentation of beta diversity after the intervention in the PRO + VIT D group (*p* = 0.0005) and an extended time to exhaustion to 559.00 ± 68.99; compared to the value before (496.30 ± 89.98; *p* = 0.023) after combined probiotic and vitamin D_3_ supplementation in MMA athletes. No effect was observed in the VIT D group.

**Conclusion:**

Our results indicate that combined treatment of probiotics and vitamin D_3_ may cause alterations in alpha and beta diversity and the composition of the gut microbiota in MMA athletes. We observed an improvement in epithelial cell permeability and an extended time to exhaustion during exercise in MMA athletes following a 4-week combined probiotic and vitamin D_3_ treatment.

## 1. Introduction

The human intestinal microbiome is inhabited by approximately 10^14^ microorganisms and has been recognized as one of the most complex sites in the human body (1). The most abundant population is bacteria. It seems that some bacterial species may affect the nutritional status of the host, metabolic pathways, and the immune system function and predominantly contribute to maintaining the integrity of epithelial cells (2). Moreover, the intestinal microbiome may indirectly affect physiological adaptations during the training process. This phenomenon is called the gut-muscle axis and is based on the assumption that certain microbes can impact muscle function (3).

Moderate physical activity has a positive influence on human gut microbiome composition, especially in the field of the diversity of bacteria species as well as bacteria genes involved in carbohydrate and protein metabolism and production of short-chain fatty acids (SCFA) (4, 5). However, training overload may disturb the homeostasis among intestine microbes, which is particularly evident in professional athletes (6). Specifically, deterioration of intestinal blood perfusion caused by high-volume exercises leads to temporary gastrointestinal tract ischemia as well as gut mucous barrier dysfunction (7). As a consequence, increased intestinal permeability occurs, referred to in the literature as “leaky gut” (8). In this scenario, negative changes in gut microbes profile are observed, promoting the growth of potentially harmful bacteria such as *Peptostreptococcus*, *Staphylococcus*, *Peptoniphilus*, *Acidaminococcus*, and *Fusobacterium* instead of bacteria species producing anti-inflammatory mediators including *Bacteroides*, *Faecalibacterium*, *Collinsella,* and *Roseburia* (9). Moreover, bacteria and their associated toxins translocate into the bloodstream, leading to the exacerbation of both local and systemic inflammatory responses (10). It is clear that chronic inflammation and oxidative stress increase catabolism, negatively impact regeneration processes, and lead to a decline of muscle function (3). Therefore, strategies aimed at enhancing regeneration and decreasing inflammatory response are important for professional athletes.

Mixed Martial Arts (MMA) is characterized as an interval sport, where high-intensity actions during the fight are interspersed with low-intensity actions or short breaks. Therefore, during MMA training, anaerobic pathways exceed aerobics and constitute the main source of energy (11). Thus, the MMA training program focuses on both aerobic and anaerobic capacity enhancements.

In a previous review, we described that the gut-muscle axis is associated with the modulation of inflammatory pathways, oxidative stress, anabolic and catabolic processes, glucose metabolism, mitochondrial function, and central nervous system health. All of these affect maximal oxygen uptake, muscle function, and training adaptation (3). Furthermore, numerous studies have provided evidence that probiotic intake may reduce inflammatory response as well as improve antioxidant potential (12, 13). In some cases, it is related to improved athletic performance (14–16). Moreover, certain data have demonstrated the benefits of some bacteria strains on sports performance *via* the improvement of gut homeostasis and intestinal permeability. It has been shown that a formula containing *Bifidobacterium bifidum* W23, *Bifidobacterium lactis* W51, *Lactobacillus acidophilus* W22, *Levilactobacillus brevis* W63, *Lactococcus Lactis* W58 decreased zonulin in feces and improved the exercise-induced inflammatory state in trained men (17). According to the stance of the International Society of Sports Nutrition on probiotics, probiotic intake is linked to a multitude of health benefits. Probiotic supplementation has been described as contributing to promoting a healthy immune response, improving recovery and responses to physical or mental stressors, reducing lactate levels, and increasing neurotransmitter synthesis (18). Additionally, the positive effect of certain bacterial strains may be enhanced by vitamin D_3_, the deficiency of which is observed widely in the Polish population and affects 85% of Poles (19). The detection of the vitamin D receptor (VDR) in skeletal muscle has provided evidence that highlights the beneficial effects of cholecalciferol on muscle metabolism. Therefore, supplementation of protective doses of vitamin D_3_ is necessary for athletes’ health and performance. In our previous study, it has been shown that combined probiotics with vitamin D_3_ supplementation improved the lactate utilization ratio, total work, and average power obtained during the anaerobic in the MMA athletes (20). Therefore, the present study is intended to cover the remaining objectives of the project and answer the question of whether combined probiotics with vitamin D_3_ ingestion can modify the composition of the gut microbiome and epithelial cell permeability, influence the inflammatory response, and ultimately improve aerobic capacity. The purpose of the study is to provide the evidence and answer the question if combined probiotics and vitamin D_3_ may enhance sport performance and muscle health in professional athletes. Moreover, we try to explain potential mechanisms of action through, which intestinal homeostasis can support the exercise capacity of athletes.

## 2. Materials and methods

### 2.1. Study design

As described in our previously published study, the parallel study was a double-blind, placebo-controlled clinical trial (20). Athletes were randomly divided into the groups receiving a multistrain probiotic mixture and vitamin D_3_ (PRO+VIT D) or vitamin D_3_ (VIT D) receiving a placebo instead of probiotics and vitamin D_3_. Vitamin D deficiency commonly occurs in the Polish population and is found in 85% of Poles (19); therefore, we decided to supplement both groups with vitamin D_3_. All study procedures were performed twice: before and immediately after 4 weeks of supplementation. In accordance with the Declaration of Helsinki, the project has been approved by the Independent Bioethics Committee (No. NKNNB/643/2019–2020) and was registered in Clinical Trials under the identifier NCT04759729. We adhered to the Standard Protocol Items: Recommendations for Interventional Trials (SPIRIT) (21, 22).

#### 2.1.1. Participants

A total of 25 male athletes who were well trained in MMA were initially enrolled in the study. The participants were recruited from Gdansk, Poland, and the surrounding areas. They were actively involved in typical mixed martial arts workouts that encompassed various disciplines such as kickboxing, Brazilian jiu-jitsu, and wrestling practice and included endurance and strength training sessions. Regarding the inclusion criteria, participants were required to have a minimum of 3 years of MMA training experience, participation in at least 3 fights, and a minimum of five training sessions per week. On the other hand, individuals with a history of inflammatory bowel diseases, heart failure, recent antibiotic therapy within the past 2 months, or chronic injuries within the last 6 months were excluded from the study. Previous research has highlighted that there are differences in gut microbiome composition related to gender and age (23, 24). To minimize data variability, we exclude females and subjects under the age of 18.

#### 2.1.2. Intervention

We used a probiotic mixture composed of lyophilized strains of bacteria: *Bifidobacterium lactis* W51, *Levilactobacillus brevis* W63, *Lactobacillus acidophilus* W22, *Bifidobacterium bifidum* W23, and *Lactococcus lactis* W58. This probiotic mixture was combined with maize starch, maltodextrin, and plant proteins and coated with hydroxypropyl ethylcellulose tablets. The probiotic mixture is commercially known as Sanprobi® Active & Sport and is produced in Szczecin, Poland. Detailed product characteristics, which were consistent with a previously published study (20), have been shown in Supplementary Data. Besides probiotics, athletes from both VIT D and PRO+VIT D groups received 5 mL of Vitamin D_3_ supplement (in oil) containing 0.5 mg of Cholecalciferol per 1 mL, which encompasses 20,000 IU, and Miglyol 812 as an excipient (Juvit D3). Participants were instructed to supplement 3,500 IU (3–4 drops) daily during the intervention period. Athletes reported no adverse events and adhered to taking the supplements during the intervention.

#### 2.1.3. Study protocol

**Figure 1:**
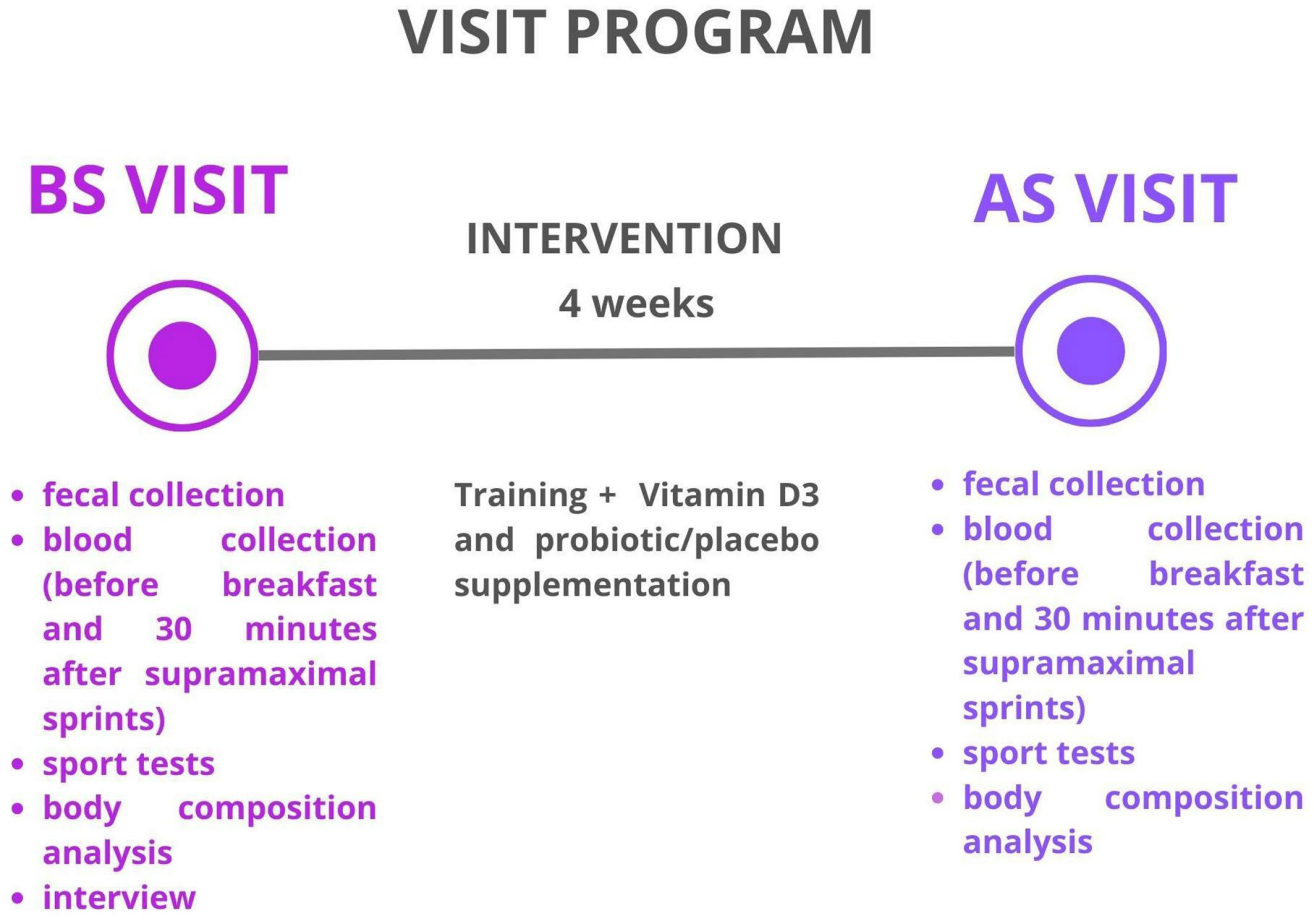
Visit programme.

The participants underwent two examinations: the first was conducted BS during the baseline visit, and the second was conducted AS at the follow-up visit. During both evaluations, the athletes completed assessments of body composition, a 3-day nutritional interview, and a cardio-respiratory fitness evaluation. Additionally, fecal samples and blood samples were collected before and after a sport test at both the BS and AS visits to assess specific parameters of inflammation. Participants were instructed to maintain their regular nutrition habits and training program throughout the study. Moreover, the athletes were advised not to make any changes to their training or dietary habits during the study period to minimize the risk of external factors influencing the collected data. All participants were required to engage in a minimum of 5 typical MMA training sessions per week, which included a combination of standing combat, grappling, ground fighting, and striking, as well as strength and endurance exercises. The average duration of these training sessions ranged from 60 to 90 min. A visit program is shown in Figure 1.

#### 2.1.4. Background information, diet, and training assessment

During the baseline visit, background information was gathered from the athletes. They were asked about their past and present injuries, diseases, medical procedures, as well as any mental or physical problems they may have experienced. Additionally, the athletes were questioned about their dietary habits and current use of supplements. Body composition analysis was also conducted during these interviews. The same interviews and assessments were performed both before and after the intervention (BS and AS). The specific procedures for collecting this information and conducting the assessments are shown in Supplementary Data.

### 2.2. Assessments and data collection

#### 2.2.1. Aerobic fitness assessment

The cardiopulmonary exercise test (VO_2_ max test) was applied in the two-time points: baseline and follow-up visit, in the morning, after a balanced breakfast (60 g of wheat bread, 60 g of strawberry jam, and 60 g of banana). Athletes were instructed not to make any physical effort 1 day before testing. To determine aerobic capacity, participants performed a graded cycle ergometry (ViaSprint 150P, Ergoline, Germany) test. Before the examination, the bicycle saddle was individually adjusted to obtain the athletes position with the slightly bent knee in the lowest pedal location to prevent hyperextension of the limbs during the test. Participants were allowed a 5-min warm-up at an intensity of 50 W with a pedaling cadence of 60 rpm. Immediately after the warm-up, the participants began cycling, in which resistance was increased every 3 min by 50 W until the subjects reached the point of volitional exhaustion. Breath by breath pulmonary gas exchange was measured by an Oxycon-Pro analyzer (Jaeger Oxycon Champion, Viasys Healthcare GmbH, Germany). Heart rates were monitored continuously by telemetry (Polar Monitors, Electro, Kempele, Finland). Maximal heart rate (HRmax), maximal respiratory exchange ratio (RER), and maximal aerobic power (MAP) were calculated at the VO_2_ max level.

#### 2.2.2. Venous blood collection

The blood samples were collected at two points time: before breakfast and 30 min after supramaximal sprints (Wingate anaerobic test based), as described in the Supplementary Data (20). Blood samples from the antecubital vein (v. mediana cubiti) were collected by a professional nurse into appropriate standardized tubes containing clot activator. Each time, up to 4 ml of blood was collected. The blood samples were centrifuged at 3000 × *g* to separate the serum. The material was placed into separately labeled microcentrifuge tubes and stored at −80°C for later determination. We assessed the level of pro- (IL-6 and TNF-α) as well as anti-inflammatory cytokines (L-2 and IL-15) using a commercially available enzyme-linked immunosorbent assay (ELISA) kits Diaclone SAS, Company of Medix Biochemica Group, France (No. 950.035.192, 950.090.192, 850.870.192, and 873.000.192, respectively) according to the “manufacturer’s instructions.”

#### 2.2.3. Fecal collection

The subjects were provided a fecal sample indirectly before and after the intervention period. The material was collected by participants into a special standardized container. Subjects received adequate instructions for collecting and handling the material. Stool samples were immediately frozen and stored at – 80°C. Collected fecal samples were subjected to quantitative and qualitative content of the intestinal microbiota using the new generation sequencing method (NGS).

#### 2.2.4. New generation sequencing

Metagenomic DNA was sequenced using Illumina 2x151bp shallow shotgun sequencing approach (25). Initial quality control, adapter removal, and merging of paired-end reads were performed using a self-learning pipeline SHI7 (26). Taxonomic profiling was performed using a shallow-shotgun computational pipeline SHOGUN (10.1093/bioinformatics/btaa277) using pipeline command with a bowtie2 aligner. The composition of the microbiome was characterized primarily by alpha and beta diversity. The diversity of microbial communities in one sample (alpha) was measured using several indicators, such as Chao1, ACE, Shannon, and inverted Simpson. All indicators were calculated based on species level data (without any pre-processing, e.g., removal of rare species) after rarefying to an even sequencing depth of 23,424. The diversity of microbial communities between samples (beta) was measured using the Bray-Curtis distance calculated on genus level after rarefying to an even sequencing depth of 23,137. Both rarefactions were done using the rtk R package (version 0.2.6.1). Permutation multivariate analysis of variance (PERMANOVA) with strata (to block by individual) was used to assess the significance of the change in microbiota composition during intervention. Collected fecal samples were analyzed in the context of the presence of intestinal permeability parameters such as calprotectin and zonulin, as well as bacterial metabolism products such as short chain fatty acids (SCFA).

#### 2.2.5. SCFA analyses

The synthesis of SCFA was done using gas chromatography with the Agilent Technologies 1,260 A GC system with a Flame Ionization Detector (FID). A silica capillary column with a free fatty acid phase (DB-FFAP, 30 m × 0.53 mm × 0.5 mm) was used. Hydrogen was supplied as a carrier gas at a flow rate of 14.4 ml/min. The initial temperature was 100°C. It was held for 0.5 min, then raised to 180°C at a rate of 80°C/min and held for 1 min. The temperature was then increased to 200°C (20°C/min) and finally held at 200°C for 5 min. The injection volume was 1 μl and the duration of each analysis was approximately 17.5 min. SCFAs were identified qualitatively by comparing the retention times to a standard, namely 2-ethyl butanoic acid. For quantitative analysis, ChemStation Software (Agilent Technologies, UK) is used. The concentrations of individual acids were converted according to the internal standard.

#### 2.2.6. Gut barrier integrity parameters

Gut barrier integrity markers: zonulin and calprotectin were evaluated using commercially available ELISA kits (Immunodiagnostic AG, Bensheim, Germany; No K5601 and K6927, respectively). The procedures followed the manufacturers’ instructions.

### 2.3. Statistical analysis

The statistical analysis of all raw data was conducted using the software program Statistica 13.3 (StatSoft Inc., Tulsa, OK, USA) and R (R Core Team, 2023). R: A language and environment for statistical computing. R Foundation for Statistical Computing, Vienna, Austria.^1^ Only complete data from participants who completed all intervention periods and study procedures (*n* = 23) were included in the analysis. Before analysis, the data were assessed for normality using the Shapiro–Wilk W-test. Descriptive statistics, including mean values with 95% confidence intervals, were utilized to examine trends in the analyzed parameters and provide background information. Statistical analysis was performed using a two-way ANOVA test and general linear mixed-effect models (lme4 R package). Following ANOVA, to determine statistical significance, post-hoc testing for specific differences was conducted using Tuckey’s Honestly Significant Difference (Tukey’s HSD) method. To compare predictions made by linear mixed-effects models for different regressor values (average marginal effects), the function *comparison* from the R marginal effects (0.12.0) package was used. The threshold for statistical significance was set at *p* < 0.05.

## 3. Results

The study initially enrolled a total of 25 MMA athletes, but two athletes from the PRO+VIT D did not complete the protocol, more details related to MMA athletes were partially presented by Przewłócka et al. (20). There were no statistically significant anthropometric differences between groups at the baseline visit. The characteristics of MMA athletes are shown in Table 1.

**Table 1:** Participants characteristics.

Participants’ information	VIT D Mean ± SD	PRO+VIT D Mean ± SD
Age	26.02 ± 4.00	24.70 ± 6.50
Height (cm)	179.30 ± 7.70	182.20 ± 9.30
Weight (kg)	80.20 ± 9.80	81.10 ± 12.00
FFM (kg)	71.94 ± 8.20	73.61 ± 9.30
Vit D BS [ng/ml]	29.56 ± 12.62	24.89 ± 9.68
Vit D AS [ng/ml]	30.63 ± 11.66	26.07 ± 10.39
HR max	185.00 ± 10.73	187.00 ± 11.34
Years of training	10.10 ± 4.40	9.90 ± 4.00
Quantity of training (hours/week)	11.40 ± 3.10	11.80 ± 3.40

FFM, fat-free mass; BS, before supplementation; AS, after supplementation; SD, standard deviation; HR max, maximal heart rate obtained during VO_2_ max test.

### 3.1. Effects of supplementation on aerobic performance

Analyzing the effect of PRO + VIT D supplementation on aerobic capacity parameters, significant differences observed in exercise time to exhaustion were found before supplementation (BS) compared to after supplementation (AS); *p = 0.023. There were no statistically significant differences in the VIT D group (*p* = 0.685). We found no differences between groups in maximal oxygen uptake value (VO_2_ max), maximal aerobic power (MAP) as well as maximal respiratory exchange ratio (RER) during the VO_2_ max test (Table 2).

**Table 2:** Results obtained during aerobic fitness assessment.

Variable	VIT D			PRO+VIT D		
	BS Mean ± SD	AS Mean ± SD	Value of *p*	BS Mean ± SD	AS Mean ± SD	Value of *p*
RER [vCO_2_/vO_2_]	1.19 ± 0.10	1.19 ± 0.06	0.999	1.21 ± 0.08	1.19 ± 0.05	0.869
VO_2_ max [ml/min/kg^−1^]	52.33 ± 5.06	52.48 ± 3.76	0.999	56.92 ± 0.83	56.37 ± 7.09	0.969
MAP [W]	306.82 ± 33.70	311.36 ± 40.87	0.940	317.50 ± 45.72	330.00 ± 42.16	0.096
Time [s]	489.91 ± 72.02	468.55 ± 102.03	0.668	496.30 ± 89.98	559.00 ± 68.99	0.023*

RER, respiratory exchange ratio; VO_2_ max, maximal oxygen uptake; MAP, maximal aerobic power; Time, exercise to exhaustion time; BS, before supplementation; AS, after supplementation; SD, standard deviation; Statistical significant (*p < 0.05).

### 3.2. Effects of supplementation on inflammatory state

No statistically significant differences were observed in the serum concentrations of IL-2, IL-6, IL-15, and TNF-α between the VIT D and PRO+VIT D groups after the supplementation period. We found a statistically significant increase in serum IL-6 concentration after exercise in both groups, both before and after supplementation (BS – *p* < 0.001 in both groups, AS – *p* = 0.033 in the PRO+VIT D; *p* = 0.029 in the VIT D group). Similarly, it was observed a significant increase in serum IL-15 concentration after intervention in the VIT D group (*p* = 0.038). However, the pairwise analysis did not show statistically significant changes between groups. Predicted outcomes based on fitted linear mixed-effects models are presented in Table 3.

**Table 3:** Average marginal effects as a difference in predicted outcomes (Before workout versus After workout) for the combination of levels of supplementation and intervention.

Outcome	Time	Intervention	*E*st	SE	*P*	*E*st_pairwise_	SE_pairwise_	*P*_pairwise_
IL2	BS	VIT D	−0.008	0.056	0.888	0.024	0.077	0.757
	BS	PRO+VIT D	0.016	0.053	0.763			
	AS	VIT D	0.022	0.056	0.693	0.004	0.077	0.962
	AS	PRO+VIT D	0.026	0.053	0.631			
IL6	BS	VIT D	1.26	0.35	<0.001**	−0.02	0.48	0.975
	BS	PRO+VIT D	1.24	0.33	<0.001**			
	AS	VIT D	0.76	0.35	0.029*	−0.05	0.48	0.915
	AS	PRO+VIT D	0.71	0.34	0.033*			
IL15	BS	VIT D	−0.64	1.51	0.675	2.04	2.09	0.330
	BS	PRO+VIT D	1.40	1.45	0.332			
	AS	VIT D	3.13	1.51	0.038*	−3.86	2.09	0.065
	AS	PRO+VIT D	−0.73	1.45	0.616			
TNF-α	BS	VIT D	168.2	108.6	0.122	−201.8	147	0.170
	BS	PRO+VIT D	−33.6	99.2	0.734			
	AS	VIT D	68.0	108.6	0.531	−83.1	147	0.572
	AS	PRO+VIT D	−15.2	99.2	0.878			

Est, estimates; SE, standard error; statistically significant (**p < 0.001); statistically significant (*p < 0.05). BS, before supplementation; AS, after supplementation.

### 3.3. Effects of supplementation on microbiome profile

**Figure 2:**
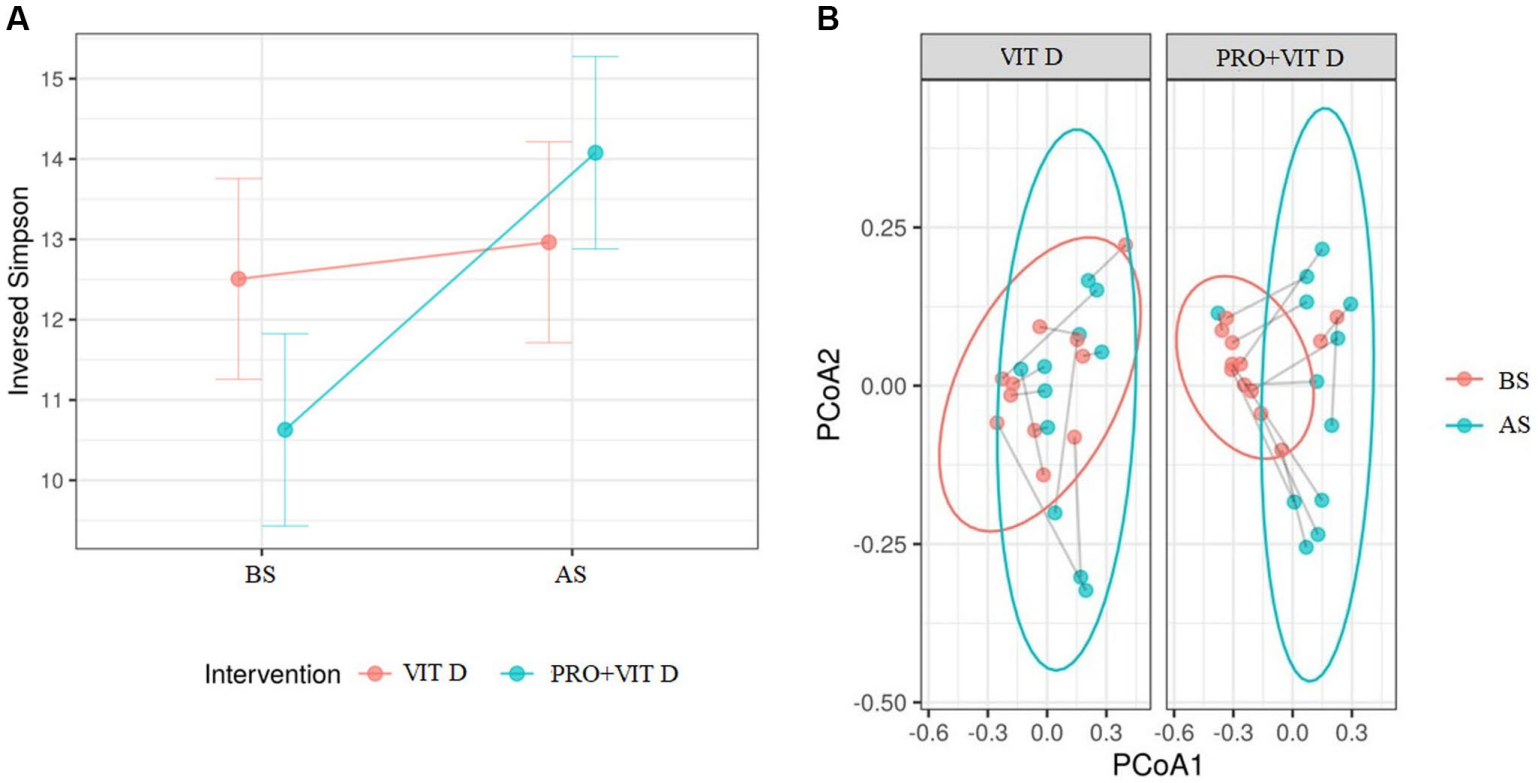
**(A)** The comparison of changes in alpha diversity between groups as a result of the intervention (*p* = 0.086), FDR adjusted value of *p* (*Q*) = 0.166. **(B)** The comparison of changes in beta diversity measured *via* Bray–Curtis between groups as a result of the intervention (***p = 0.0005). BS, before supplementation; AS, after supplementation.

Analysis of the gut microbiome was performed using a general linear mixed-effects model, where the time (BS and AS) of the intervention (PRO + VIT D or VIT D) interactions were examined. The predictor effect plot indicated that the interaction was significant because the alpha-diversity increase in the probiotic group was greater, but the baseline (PRE) values were lower, so the follow-up (POST) values between interventions were very similar. In addition, following FDR adjustment, none of the value of *p*s remained statistically significant. Beta diversity measured by Bray-Curtis showed statistically significant shifts in microbiota composition during the intervention in the PRO+VIT D group (*p* = 0.0005), but there were no significant changes in the VIT D group (*p* = 0.145, Figure 2).

**Figure 3:**
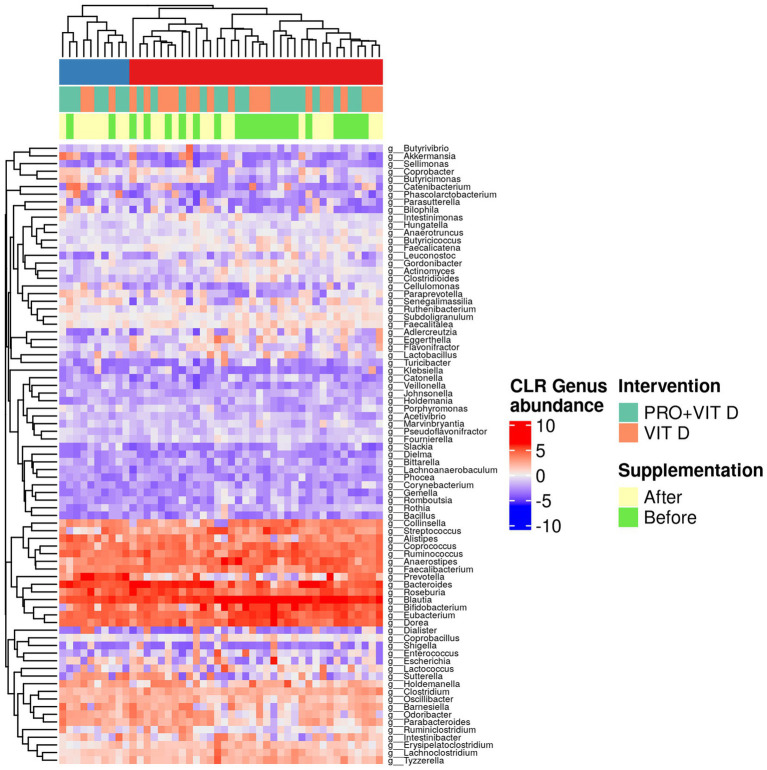
Heatmap of the abundance of the gut microbiota at the genus level center-log transformed (CLR) among each group.

Moreover, we observed significant changes in the gut microbiome composition after probiotics supplementation, when individual taxa were considered. A total change in bacteria profile and the abundance of the gut microbiota at the genus level among each group is presented in Figure 3. Our results indicate that supplementation significantly affected intestinal microbes’ profile and contributed to the growth of bacteria having a potentially beneficial effect on the host health (e.g., Bacteroides genus, *Roseburia inulinivorans*, *Prevotella* genus, Lactobacillaceae family). We found a significant growth of Negativicutes class in the PRO+VIT D group (Est = 1.98, *p* = 0.006), but not in the VIT D group (Est = −0.25, *p* = 0.738). The changes in the selected bacteria are presented in Table 4. The total observed changes are shown as Supplementary Data.

**Table 4:** Total abundance of selected bacteria before and after 4 weeks of probiotics supplementation.

Outcome	Intervention	*E*st	SE	*P*	*E*st_pairwise_	SE_pairwise_	*P*_pairwise_
*Bacteroides fluxus*	VIT D	0.08	0.48	0.859	2.02	0.66	0.002**
	PRO+VIT D	2.11	0.46	<0.001			
*Lachnospiraceae bacterium*	VIT D	−0.28	0.27	0.309	−1.27	0.38	<0.001**
	PRO+VIT D	−1.55	0.26	<0.001			
*Roseburia inulinivorans*	VIT D	−0.66	0.36	0.065	1.40	0.49	0.005**
	PRO+VIT D	0.74	0.34	0.030			
*Peptostreptococcaceae bacterium*	VIT D	0.84	0.68	0.222	−2.69	0.95	0.004**
	PRO+VIT D	−1.86	0.65	0.005			
*Bacteroides* genus	VIT D	0.30	0.67	0.657	2.29	0.92	0.013*
	VIT D	0.30	0.67	0.657			
*Collinsella* genus	VIT D	0.37	0.46	0.413	−1.65	0.63	0.009**
	PRO+VIT D	−1.28	0.44	0.003			
*Faecalibacterium* genus	VIT D	−0.19	0.35	0.601	1.21	0.49	0.013*
	PRO+VIT D	1.03	0.34	0.002			
*Prevotella* genus	VIT D	0.62	0.96	0.516	−1.01	0.33	0.002**
	PRO+VIT D	3.62	0.92	<0.001			
Lactobacillaceae family	VIT D	0.88	0.62	0.158	−2.04	0.86	0.018*
	PRO+VIT D	−1.17	0.60	0.050			
Negativicutes class	VIT D	−0.25	0.75	0.738	2.23	1.04	0.032*
	PRO+VIT D	1.98	0.72	0.006			
Firmicutues class	VIT D	−0.75	0.32	0.021	−0.93	0.45	0.038*
	PRO+VIT D	−1.68	0.31	<0.001			

Est, estimates; SE, standard error; Statistically significant (**p < 0.01). statistically significant (*p < 0.05).

### 3.4. Effects of supplementation on SCFA

The analysis of the percentage changes in selected SCFA concentrations showed no significant differences between PRO + VIT D and VIT D groups. We noted that propionate decreased after supplementation in both groups; however, this decrease was greater in the VIT D group. This trend was close, but not statistically significant (*p* = 0.061). The total changes in the SCFA profiles are presented in Table 5.

**Table 5:** Short-chain fatty acids – average marginal effects as a difference in predicted outcomes (BS versus AS) for PRO + VIT D and VIT D groups.

Outcome	Intervention	Est	SE	*z*	*P*	*E*st_pairwise_	SE_pairwise_	*P*_pairwise_
C2:0 (%)	VIT D	−0.39	2.57	−0.15	0.879	−3.19	3.51	0.364
	PRO + VIT D	−3.58	2.39	−1.50	0.134			
C3:0 (%)	VIT D	−2.30	0.81	−2.86	0.004	2.07	1.10	0.061
	PRO + VIT D	−0.23	0.75	−0.31	0.756			
C4n (%)	VIT D	2.26	2.04	1.11	0.266	1.20	2.79	0.668
	PRO + VIT D	3.46	1.90	1.82	0.069			
C5n (%)	VIT D	−0.21	0.21	−1.01	0.311	0.13	0.29	0.650
	PRO + VIT D	−0.08	0.20	−0.43	0.670			

C2:0-acetic acid; C3:0 – propionic acid; C4:0- butyric acid; C5:0 – Valeric acid; Est, estimates; SE, standard error; z, Est/SE.

### 3.5. Effects of supplementation on intestinal permeability parameters

**Figure 4:**
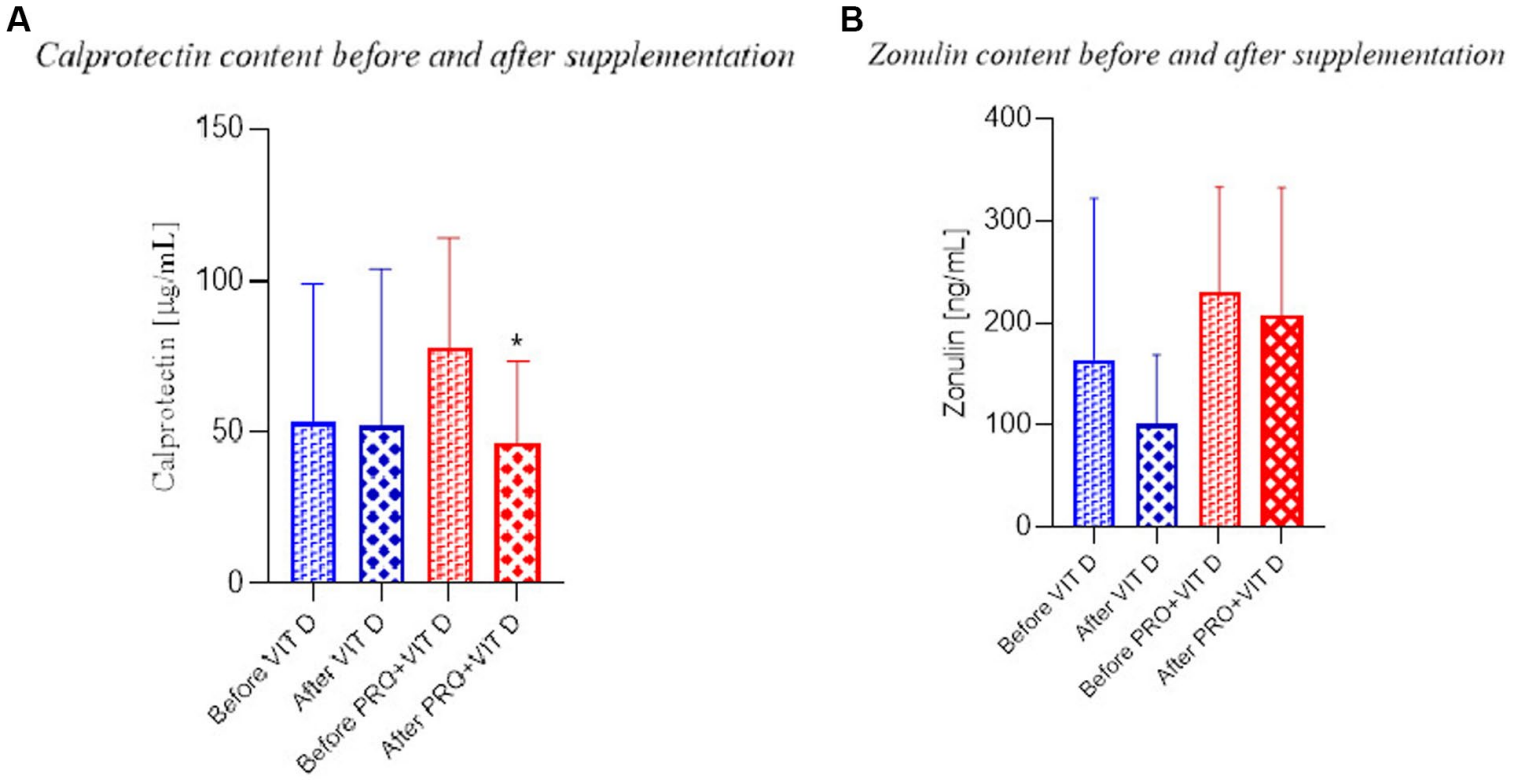
Calprotectin and zonulin content before and after supplementation **(A)** Calprotectin Statistical significant (^*^p < 0.05) BS vs. AS in the group PRO+VIT D; **(B)** Zonulin the lack of changes in both groups.

The fecal zonulin and calprotectin concentrations in both groups before supplementation were not significantly different. After 4-week of supplementation, we found a significantly lower concentration of calprotectin in the PRO+VIT D group (34.79 ± 24.38 mmol/L) compared to the value before (69.50 ± 46.91) supplementation (*p* = 0.030; Figure 4A). No significant effects were observed in the VIT D group. We did not observe significant differences in the fecal concentrations of zonulin in both groups (Figure 4B).

## 4. Discussion

To the best of our knowledge, this is the first study to comprehensively assess the influence of a combined multistrain probiotic mixture and vitamin D_3_ supplementation in a Mixed Martial Arts (MMA) athlete population. The current study examined the influence of a 4-week supplementation on aerobic performance, inflammatory state, gut microbiome composition, and intestinal permeability. We found that the 4-week treatment positively affected the gut microbiome profile measured *via* inverted Simpson distance and Bray-Curtis distance, as well as the abundance of certain bacteria. The intervention positively correlated with the total time to exhaustion obtained in the aerobic test; however, it did not affect the maximal oxygen uptake and lactate threshold. Probiotic supplementation also improved intestinal permeability. Our results suggest that the combined supplementation of a multistrain probiotic mixture with vitamin D_3_ improved physical performance and was associated with significant changes in the gut microbial profile. Therefore, combined probiotic and vitamin D_3_ supplementation may be considered a strategy for promoting exercise performance among competitive MMA athletes.

Recently, it was reported that the gut microbiota profile plays a crucial role in immune function (18, 27, 28) and brain health (29, 30), which may be indirect factors influencing physiological adaptation to training. However, the potential beneficial connection between intestinal microbiota composition, muscle function, and exercise performance is not clearly understood. Increasing evidence has confirmed the importance of the interplay between gut homeostasis, inflammatory processes, and skeletal muscle adaptation to training, as demonstrated in a previous review (3).

The MMA training program focused on both aerobic and anaerobic capacity enhancements. Increased ATP resynthesis *via* oxidative phosphorylation during submaximal exercise enhances sports performance by improving LA utilization as well as pyruvate oxidation (11). In the present study, we observed a significant improvement in time to exhaustion during the VO_2_ max test, reflecting endurance capacity but not the VO_2_ max value. Recent studies indicated that VO_2_ max may increase mainly as a result of an effective training program that includes interval sessions (31, 32). Moreover, training programs more effectively influence VO_2_ max than any nutritional strategy. According to the literature, certain nutritional strategies do not significantly elevate oxygen uptake in the athletic population (33, 34). In our study, athletes were instructed to maintain their MMA training program. It is important to note that typical MMA training does not specifically target to improvement VO_2_ max. Consequently, the increase in total time to exhaustion observed in our study was not associated with changes in VO_2_ max value, instead, it was likely attributed to alterations in the gut microbiome profile. Some bacteria may enhance physical performance through a microbial-encoded enzymatic process, facilitating LA utilization while also providing substrates for gluconeogenesis and additional energy.

We found that athletes who were supplemented with a multistrain probiotics mixture combined with vitamin D_3_ extended their exercise to exhaustion time. Our results are in line with those of a study conducted by Huang et al., who supplemented triathletes with *Lactobacillus plantarum* PS128. The authors showed that the intervention improved endurance running performance through intestinal microbiome alteration but did not affect maximal oxygen uptake (14). A similar effect was observed by Lin et al. after 5 weeks of *Bifidobacterium longum subsp. longum* OLP-01 supplementation period in the well-trained middle- and long-distance runners. Researchers detected that the change in the 12-min Cooper’s test running distance significantly increased, as well as the total abundance of the gut microbiota (35). Roberts et al. obtained similar results after 12 weeks of supplementation of the probiotic mixture containing ed. *Lactobacillus* and *Bifidobacterium* species. In the group of recreative training adults, improved certain stage time of triathlete race and endotoxemia were a result of probiotic intake (36). Scheiman et al. reported that mice supplemented with *Veilonella atypical* showed improvement in extended exercise to exhaustion time as well as higher LA utilization levels (37). Similarly, animals supplemented with *Bacteroides fragilis* showed improved extended exercise-to-exhaustion time (38). These data support the hypothesis that probiotic intake may have a positive effect on endurance capacity through the alteration of gut microbes; however, the influence on VO_2_ max is limited. In our previous study, we demonstrated that a 4-week combined supplementation of a multistrain probiotic and vitamin D_3_ resulted in a significant increase in the rate of lactate (LA) utilization among MMA athletes after supramaximal sprints (20). The efficient metabolism of LA is of utmost importance for athletes’ sports performance. It is widely recognized that the accumulation of LA within muscle tissues and the subsequent decrease in pH levels among muscle cells contribute to the onset of fatigue during training. These effects are primarily attributed to the detrimental impact on glycolytic energy production and the release of potassium ions (39). Consequently, we posit that probiotics, although limited in their impact on maximal oxygen uptake, may enhance endurance capacity through favorable modulation of the gut microbiome profile, potentially leading to improve LA utilization.

In contrast to our results, there are some studies indicating no effect on aerobic capacities after certain probiotics supplementation. In the study conducted by Carbuhn et al., 12 weeks of *Bifidobacterium longum* did not affect aerobic swim performance in female swimmers. However, in this study, the gut microbiome composition was not assessed, thus, it is not known whether the intervention affected the composition of the intestinal bacteria profile. The number of researchers evaluating the influence of probiotic supplementation on the gut microbiome composition in athletes is limited. Therefore, more research is needed in this area to explore the potential mechanism(s) *via* certain bacteria species that improve endurance capacity in athletes.

One of the most known mechanisms by which the gut microbiome may affect sports performance is their ability to alter the inflammatory response. It is well established that probiotics may suppress intestinal inflammation by down-regulation of Toll Like Receptors (TLR) expression (40) as well as enhanced innate immunity *via* different mechanisms, like upregulation of immunoglobulins, antimicrobial proteins, phagocytic activity, natural killer cells activity, and T and B lymphocytes function improvements (18). Microbial ability to SCFA production and thus enhance the integrity of intestinal mucus is described as a potential mechanism protecting against excessive activation of the immune system and hence maintaining appropriate pro- and anti-inflammatory cytokines ratio (41). It was shown that chronic inflammation, manifested in pro-inflammatory cytokines overproduction, may disrupt regeneration processes and inhibit muscle protein synthesis and metabolic adaptations to training (42, 43).

In our study, we did not observe any significant changes of pro- as well as anti-inflammatory markers in serum. Our results are not consistent with data obtained by Vaisberg et al. who demonstrated that 30 days of ingestion of fermented milk containing 40 billion of *Lactobacillus casei* Shirtota of marathon runners was able to modulate both immunological and inflammatory response. The authors found a higher level of TNF-α in serum in the placebo group and lower levels of pro-inflammatory cytokines (IL-1, IL-5, IL-6, IL-13, and TNF-α) as compared to the supplemented group after a marathon race. There were no significant changes in serum IL-6 and IL-10 between groups (13). It might have been caused by the fact that the increase of this interleukin is not solely in response to inflammation. It may also physiologically appear as a result of muscle contraction and glycogen regulation. Similarly, there was a substantial reduction of TNF-α in basketball players supplemented 12 weeks with *Bacillus subtilis* DE111, without changes in other parameters such as IL-10, zonuline, testosterone and cortisol concentration as well as sport performance parameters (12). The influence of probiotics supplementation on inflammatory response was also observed in the clinical trial conducted by Jager et al. In their study, men on resistance training supplemented with *Streptococcus thermophilus* FP4 and *Bifidobacterium breve* BR03 showed a lower IL-6 concentration up to 48 h after damaging training. Improvement of maximal voluntary isometric peak torque at 24–72 h following damaging exercises as well as flexed arm angle after the damaging workout was also shown (16). In our study, any significant changes in the level of TNF- α, IL-6, Il-2, and IL-15 were not observed. It is important to note that both groups received vitamin D3 supplementation. Therefore, it can be assumed that the treatment with vitamin D3 had a beneficial impact on reducing pro-inflammatory markers in MMA athletes. Furthermore, vitamin D3 supplementation may serve as an attenuating factor in regulating inflammation and inhibiting the immune system, making it a modifiable risk factor for reducing inflammation. This may explain why we did not observe any significant differences of pro- as well as anti-inflammatory markers in serum between the PRO+VIT D and VIT D groups. Similarly, Hoffman et al. also detected no difference in pro-inflammatory cytokines levels after *Bacillus coagulant* supplementation period among soldiers (44).

Numerous data report that an increase of microbiome diversity and a higher abundance of health-promoting bacteria species are associated with enhanced fitness (18). In our study, we observed that 4 weeks of supplementation was enough to significantly increase beta but not alpha diversity. The effect was not detected in the VIT D group. We found positive changes in the gut bacteria profile, as increase of *Bacteroides, Peptostreptococcaceae bacterium, Roseburia inulinivorans* species, and *Prevotella* genus, and decrease of potentially harmful *Lachnospiraceae bacterium*. Moreover, we detected that the *Lactobacillaceae* class augmented despite the decline of *Firmicutes* species.

The vast majority of intestinal bacterial species are *Firmicutes* and *Bacteroidetes*, thus the relative ratio between Firmicutes to Bacteroidetes (F:B) is used to describe the gut microbiota homeostasis (45). It is established that in obese individuals F:B ratio is elevated (45). However, it seems that physical training may increase bacterial species within the Firmicutes phyla. This finding was confirmed by Durk et al., who indicated that VO_2_ max was associated with an elevation in the F:B ratio among young healthy individuals (46). In the current study, we observed no differences in VO_2_ max in both groups, but we found a decrease of the F:B ratio. The combined supplementation significantly increased the total abundance of the *Bacteroides* genus and caused a reduction of the total abundance of *Firmicutes* phyla. However, some beneficial bacteria within Firmicutes phyla, e.g., *Roseburia inunilivorans, Lactobacillaceae,* and *Negativicutes* increased.

The analysis of the gut microbiome indicated a higher level of *Feacilibacterium* genus after combined intake. This bacteria was correlated with the improvement of intestinal health *via* an increase of butyrate production as well as by lowering the oxygen tension (47). The effect was confirmed by Yoonmi et al., who supplemented mice with *Feacilibacterium prausnitzi* and *Akkermansia muciniphila* (48). The authors observed recovery of the gut barrier function and increased zonulin production. In contrast, our results showed no difference in zonulin level. However, we found a reduction of fecal calprotectin – a marker of intestinal inflammation. A similar effect was found by MinAh et al., who supplemented patients with functional diarrhea with *Lactobacillus plantarum* CJLP243. The authors showed that two months of intervention resulted in a reduction of fecal calprotectin concentration (49).

Interestingly, our results displayed a significant increase of the *Collinsella* genus after combined probiotic and vitamin D3 supplementation. It was reported that this bacteria grows during a high-carbohydrate diet and was associated with improved time-trail performance by +6.5% (50). Similarly, an increase of the relative abundance of the Collinsella genus was observed by van Zanten et al., who supplemented healthy humans with synbiotics (*Lactobacillus acidophilus* NCFM and cellobiose) (51). However, the authors did not investigate any parameters of sport performance. The *Collinsella* genus is described as having favorable anti-inflammatory and immunomodulatory effects (9). Moreover, the study conducted by Kassinen et al. presented that a lower abundance of *Collinsella* genera occurs in people with irritable bowel syndrome (IBS) (52). Therefore, these bacteria may play a protective role against intestinal barrier dysfunction during stress. Furthermore, it was shown that *Collinsella* genera are associated with high blood insulin levels and have broad dietary carbohydrate metabolizing potential (50). Similarly, recent metagenomic analysis linked the growth of *Prevotella* with the increased ability of intestinal microbes to carbohydrate metabolize (53). The link between a high-fiber diet and *Prevotella* abundance was present by Kovatcheva-Datchary et al. who observed that this kind of diet resulted in the growth of the *Prevotella* genus. Moreover, the authors indicated that changes in the gut microbiome composition positively correlated with improved glucose metabolism, partially by promoting increased glycogen storage (54). Thus, we suggest that improvement in exercise performance may be related to enhancement of the efficiency of energy processes involved in carbohydrate metabolism *via* shifts in microbes engaged in glucose metabolism. However, there is a lack of studies investigating the effect of probiotic supplementation on the gut microbiome composition in MMA athletes.

Our previous published data showed that athletes who took a combined probiotic and vitamin D_3_ mixture improved lactate metabolism rate after SIE (20). Interestingly, the analysis of the gut microbiome indicated a higher abundance of *Negativicutes* class after 4 weeks supplementation period, whereas the VIT D group showed a slight decrease of this class. It is known that some human bacteria belonging to the *Negativicutes,* e.g., *Phascolarctobacterium succinatutens,* can convert succinate to propionate, and the other one, like *Veilonella* spp. convert lactate to propionate (55). The accumulation of LA and hydrogen ions in skeletal muscle and blood circulation impair physical performance due to the limitation of glycolysis and the development of fatigue during exercise (39). Thus, we suppose that *Negativicutes* class increasement might enhance endurance capacity, partially *via* improvement of lactate metabolism and thus provide additional energy. By our results, a link between members of *Negativicutes* class (*Veilonella* genus) and exercise performance was identified by Scheiman et al. Researchers observed that the relative abundance of *Veillonella* is higher in marathon runners after marathons and that inoculation of *Veilonella atypica* into mice improved exhaustive treadmill runtime (37).

One of the strengths of our study was that we not only evaluated the direct effects but also examined changes in the composition of the intestinal microbiome. We demonstrated that the intervention resulted in significant changes in the gut microbiome, which had a beneficial effect on exercise capacity. One limitation of the study was the lack of diet standardization. However, in order to minimize the potential influence of diet on the gut microbiome, athletes were instructed not to make any changes to their existing eating habits, and they were also advised to refrain from taking any medications, consuming alcohol, or smoking.

Our results indicate that combined probiotic and vitamin D_3_ treatment is beneficial for MMA athletes and may lead to shifts in both alpha and beta diversity as well as in the composition of the gut microbiota. We found a decrease in calprotectin concentration after probiotic supplementation, indicating an improvement in epithelial cell permeability. It shows that probiotics supplementation may protect athletes against intestinal inflammation. Furthermore, this supplementation extended the time to exhaustion during exercise in MMA athletes. This is a result directly indicating the benefits of supplementation with probiotics in sports, which shows that in fact, the optimization of the intestinal microbiota has a positive effect on exercise capacity. However, an effect on blood inflammatory markers and gut SCFA profiles in both groups was not observed. Our data suggest a bidirectional communication pathway between muscle cells and gut microbiota, confirming the beneficial effects of combined probiotics and vitamin D_3_ in competitive athletes.

## Data availability statement

The original contributions presented in the study are publicly available. This data can be found here: NCBI, https://www.ncbi.nlm.nih.gov/, accession number PRJNA1002235.

## Ethics statement

The studies involving humans were approved by Independent Bioethics Committee (No. NKNNB/643/2019–2020). The studies were conducted in accordance with the local legislation and institutional requirements. The participants provided their written informed consent to participate in this study.

## Author contributions

KP: Conceptualization, Formal analysis, Funding acquisition, Investigation, Methodology, Project administration, Writing – original draft, Writing – review & editing. MF: Conceptualization, Writing – review & editing. MK: Formal analysis, Writing – review & editing. KS-Ż: Formal analysis, Investigation, Writing – review & editing. JP: Investigation, Writing – review & editing. ZB: Investigation, Writing – review & editing. SK: Formal analysis, Investigation, Writing – review & editing. JK: Conceptualization, Methodology, Project administration, Supervision, Writing – original draft, Writing – review & editing.

## Footnotes

^1^ https://www.R-project.org/
