# Supplementary material for: Combined probiotics with vitamin D3 supplementation improved aerobic performance and gut microbiome composition in mixed martial arts athletes
Source: Front Nutr. 2023 Oct 11;10:1256226. doi: 10.3389/fnut.2023.1256226 (PMC10599147; doi:10.3389/fnut.2023.1256226)
Supplement: Supplementary file 1 [file Data_Sheet_1.docx]

Supplementary Material

# Supplementary Data

**Probiotics characteristics**

Probiotics mixture were composed of lyophilized strains of bacteria: *Bifidobacterium lactis* W51, *Levilactobacillus brevis* W63, *Lactobacillus acidophilus* W22, *Bifidobacterium bifidum* W23, and *Lactococcus lactis* W58. This probiotic mixture was combined with maize starch, maltodextrin, plant proteins, and coated with hydroxypropyl ethylcellulose tablets. The probiotic mixture is commercially known as Sanprobi® Active & Sport and is produced in Szczecin, Poland.

The total cell count was adjusted to 2.5 x 10^9^ colony forming units (CFU) per gram (≥ 500 million CFU in a capsule). Identical-looking capsules containing only 40 mg of maltodextrin and plant proteins were used as a placebo. The product has been tested by means of pharmacopea methods, that is culture technique. The number of CFU is guaranteed by the end of shelf life as evidenced by the use of a climatic chamber. As a placebo, we used capsules containing 40 mg of maltodextrin and plant proteins. The product has been tested by means of pharmacopea methods, that is culture technique. The number of CFU is guaranteed by the end of shelf life as evidenced by the use of a climatic chamber. Depending on the random allocation, participants obtained probiotics or placebo and were instructed to take 4 capsules daily with meals (2 capsules in the morning and two capsules in the evening). The total daily dose of 2 x 10^9^ CFU; 2 capsules in the morning and two capsules in the evening.

**Specific procedures for Background information, diet and training assessment**

Background information was gathered during the initial visit to obtain relevant details about the athletes. They were asked about their overall health, including past and current injuries, illnesses, medical procedures, and any physical or mental issues. Additionally, their use of concurrent medications was also inquired about. To determine whether habitual changes in diet or training programs could impact the gut microbiome and sports performance of athletes, a qualified sports nutritionist conducted a specific interview. The interview assessed the athletes' current food consumption, including total energy intake and nutrients derived from food, based on a 3-day food interview. The results were analyzed using Dietico, a professional food calculator, to determine the total caloric intake as well as the intake of carbohydrates, fat, and protein. Furthermore, a Food Frequency Questionnaire (FFQ) was administered. The level of physical activity was evaluated in terms of duration and frequency, considering exercise hours and the number of times per week. All athletes were required to engage in a minimum of 5 typical MMA training sessions per week, each lasting approximately 60-90 minutes. Typical MMA training encompasses a combination of standing combat, grappling, ground fighting, striking, and includes elements of strength and endurance. A specially prepared survey was used to assess the athletes' current intake of supplements. These interviews were conducted both before (BS) and after (AS) the intervention.

**Characterization of supramaximal sprints**

Anaerobic performance assessments were conducted at two specific time points the baseline visit and the follow-up visit, both taking place in the morning after consuming a balanced breakfast consisting of 80 g of wheat roll, 60 g of strawberry jam, and one banana. The testing sessions were carried out on a cycle ergometer (884E Sprint Bike, Monark, Sweden). Prior to the examination, the saddle was adjusted individually for each participant. The exercise protocol began with a 5-minute warm-up at 100 watts, which included two all-out sprints lasting 3 – 5 seconds during the last minute of the warm-up period. Subsequently, the subjects were given a 3-minute rest period for final preparation before immediately commencing the interval exercise, which consisted of three 30-second "all-out" supramaximal sprints known as the Wingate anaerobic test (WAnT). The resistance of the flywheel was set at 7.5 % of each individual's body mass and was applied at the start of the sprints. The rest periods between the cycling bouts were set to 2 minutes. The athletes were instructed to reach their maximum pedaling rate and were verbally encouraged to maintain this pace throughout the test.

# Supplementary Figures and Tables

**Table 1. Total abundance of bacteria species before and after 4 weeks probiotics supplementation**

| Outcome | Intervention | Est | SE | P | Est_pairwise_ | SE_pairwise_ | P_pairwise_ |
| --- | --- | --- | --- | --- | --- | --- | --- |
| Adlercreutzia equolifaciens | VIT D | 0.53 | 0.47 | 0.258 | -1.94 | 0.65 | 0.003 |
|  | PRO+VIT D | -1.41 | 0.45 | 0.0002 |  |  |  |
| Bacteroides fluxus | VIT D | 0.08 | 0.48 | 0.859 | 2.02 | 0.66 | 0.002 |
|  | PRO+VIT D | 2.11 | 0.46 | <0.001 |  |  |  |
| Bacteroides stercoris | VIT D | -0.18 | 0.77 | 0.820 | 2.98 | 1.07 | 0.005 |
|  | PRO+VIT D | 2.80 | 0.74 | <0.001 |  |  |  |
| Lachnospiraceae bacterium 3-1 | VIT D | -0.28 | 0.27 | 0.309 | -1.27 | 0.38 | <0.001 |
|  | PRO+VIT D | -1.55 | 0.26 | <0.001 |  |  |  |
| [Clostridium] scindens | VIT D | 0.69 | 0.50 | 0.171 | -1.98 | 0.69 | 0.004 |
|  | PRO+VIT D | -1.29 | 0.48 | 0.007 |  |  |  |
| Roseburia inulinivorans | VIT D | -0.66 | 0.36 | 0.065 | 1.40 | 0.49 | 0.005 |
|  | PRO+VIT D | 0.74 | 0.34 | 0.030 |  |  |  |
| Peptostreptococcaceae bacterium VA2 | VIT D | 0.84 | 0.68 | 0.222 | -2.69 | 0.95 | 0.004 |
|  | PRO+VIT D | -1.86 | 0.65 | 0.005 |  |  |  |
| Veillonella parvula | VIT D | 1.04 | 0.61 | 0.089 | -0.48 | 0.84 | 0.568 |
|  | PRO+VIT D | 0.56 | 0.58 | 0.342 |  |  |  |
|  |  |  |  |  |  |  |  |

**Table 2. Total abundance of bacteria genus before and after 4 weeks probiotics supplementation**

| Outcome | Intervention | Est | SE | P | Est_pairwise_ | SE_pairwise_ | P_pairwise_ |
| --- | --- | --- | --- | --- | --- | --- | --- |
| Actinomyces | VIT D | -0.48 | 0.36 | 0.183 | -1.08 | 0.50 | 0.030 |
|  | PRO+VIT D | -1.55 | 0.34 | <0.001 |  |  |  |
| Adlercreutzia | VIT D | 0.54 | 0.44 | 0.220 | -1.84 | 0.61 | 0.002 |
|  | PRO+VIT D | -1.31 | 0.42 | 0.002 |  |  |  |
| Bacteroides | VIT D | 0.30 | 0.67 | 0.657 | 2.29 | 0.92 | 0.013 |
|  | PRO+VIT D | 0.259 | 0.64 | <0.001 |  |  |  |
| Collinsella | VIT D | 0.37 | 0.46 | 0.413 | -1.65 | 0.63 | 0.009 |
|  | PRO+VIT D | -1.28 | 0.44 | 0.003 |  |  |  |
| Corynebacterium | VIT D | -0.18 | 0.36 | 0.614 | -1.01 | 0.50 | 0.042 |
|  | PRO+VIT D | -1.19 | 0.34 | <0.001 |  |  |  |
| Faecalibacterium | VIT D | -0.19 | 0.35 | 0.601 | 1.21 | 0.49 | 0.013 |
|  | PRO+VIT D | 1.03 | 0.34 | 0.002 |  |  |  |
| Faecalicatena | VIT D | -0.37 | 0.19 | 0.047 | -0.53 | 0.26 | 0.040 |
|  | PRO+VIT D | -0.89 | 0.18 | <0.001 |  |  |  |
| Lachnoanaerobaculum | VIT D | 0.81 | 0.24 | <0.001 | -1.01 | 0.33 | 0.002 |
|  | PRO+VIT D | -0.20 | 0.23 | 0.392 |  |  |  |
| Oscillibacter | VIT D | -0.60 | 0.39 | 0.126 | 1.38 | 0.54 | 0.011 |
|  | PRO+VIT D | 0.78 | 0.37 | 0.036 |  |  |  |
| Parabacteroides | VIT D | 0.83 | 0.64 | 0.195 | 1.78 | 0.89 | 0.045 |
|  | PRO+VIT D | 2.61 | 0.61 | <0.001 |  |  |  |
| Parasutterella | VIT D | 0.54 | 0.82 | 0.514 | 2.78 | 1.14 | 0.015 |
|  | PRO+VIT D | 3.32 | 0.79 | <0.001 |  |  |  |
| Prevotella | VIT D | 0.62 | 0.96 | 0.516 | 2.99 | 1.33 | 0.024 |
|  | PRO+VIT D | 3.62 | 0.92 | <0.001 |  |  |  |
| Veillonella | VIT D | 1.40 | 0.54 | 0.0095 | -0.51 | 0.75 | 0.498 |

**Table 3. Total abundance of bacteria family before and after 4 weeks probiotics supplementation**

| Outcome | Intervention | Est | SE | P | Est_pairwise_ | SE_pairwise_ | P_pairwise_ |
| --- | --- | --- | --- | --- | --- | --- | --- |
| Actinomycetaceae | VIT D | -0.49 | 0.36 | 0.171 | -1.43 | 0.49 | 0.004 |
|  | PRO+VIT D | -191 | 0.34 | <0.001 |  |  |  |
| Bacteroidaceae | VIT D | 0.25 | 0.66 | 0.710 | 2.01 | 0.91 | 0.027 |
|  | PRO+VIT D | 2.25 | 0.63 | <0.001 |  |  |  |
| Coriobacteriaceae | VIT D | 0.11 | 0.43 | 0.797 | -1.83 | 0.59 | 0.002 |
|  | PRO+VIT D | -1.72 | 0.41 | <0.001 |  |  |  |
| Corynebacteriaceae | VIT D | -0.23 | 0.36 | 0.519 | -1.29 | 0.50 | 0.009 |
|  | PRO+VIT D | -1.52 | 0.34 | <0.001 |  |  |  |
| Lactobacillaceae | VIT D | 0.88 | 0.62 | 0.158 | -2.04 | 0.86 | 0.018 |
|  | PRO+VIT D | -1.17 | 0.60 | 0.050 |  |  |  |
| Oscillospiraceae | VIT D | -0.64 | 0.38 | 0.089 | 1.1 | 0.52 | 0.034 |
|  | PRO+VIT D | -0.46 | 0.36 | 0.198 |  |  |  |
| Prevotellacea | VIT D | 0.67 | 0.93 | 0.475 | 2.63 | 1.29 | 0.042 |
|  | PRO+VIT D | 3.30 | 0.89 | <0.001 |  |  |  |
| Sutterellaceae | VIT D | 2.17 | 0.92 | 0.018 | 2.91 | 1.27 | 0.021 |
|  | PRO+VIT D | 5.08 | 0.88 | <0.001 |  |  |  |
| Veillonellaceae | VIT D | 0.42 | 0.77 | 0.586 | -0.52 | 1.06 | 0.625 |
|  | PRO+VIT D | -0.10 | 0.73 | 0.890 |  |  |  |

**Table 4. Total abundance of bacteria order before and after 4 weeks probiotics supplementation**

| Outcome | Intervention | Est | SE | P | Est_pairwise_ | SE_pairwise_ | P_pairwise_ |
| --- | --- | --- | --- | --- | --- | --- | --- |
| Actinomycetales | VIT D | -0.58 | 0.31 | 0.064 | -1.32 | 0.44 | 0.002 |
|  | PRO+VIT D | -1.90 | 0.30 | <0.001 |  |  |  |
| Burkholderiales | VIT D | 2.07 | 0.91 | 0.023 | 2.90 | 1.26 | 0.022 |
|  | PRO+VIT D | 4.97 | 0.87 | <0.001 |  |  |  |
| Coriobacteriales | VIT D | 0.002 | 0.38 | 0.006 | -1.70 | 0.53 | 0.001 |
|  | PRO+VIT D | -1.70 | 0.36 | <0.001 |  |  |  |
| Corynebacteriales | VIT D | -0.01 | 0.37 | 0.969 | -1.51 | 0.52 | 0.003 |
|  | PRO+VIT D | -1.52 | 0.36 | <0.001 |  |  |  |
| Veillonellales | VIT D | 0.32 | 0.75 | 0.664 | -0.41 | 1.03 | 0.693 |
|  | PRO+VIT D | -0.08 | 0.71 | 0.907 |  |  |  |

**Table 5. Total abundance of bacteria class before and after 4 weeks probiotics supplementation**

| Outcome | Intervention | Est | SE | P | Est_pairwise_ | SE_pairwise_ | P_pairwise_ |
| --- | --- | --- | --- | --- | --- | --- | --- |
| Bacilli | VIT D | -0.21 | 0.57 | 0.713 | -1.67 | 0.79 | 0.035 |
|  | PRO+VIT D | -1.87 | 0.55 | <0.001 |  |  |  |
| Clostridia | VIT D | -0.66 | 0.34 | 0.053 | -1.28 | 0.47 | 0.007 |
|  | PRO+VIT D | -1.93 | 0.33 | <0.001 |  |  |  |
| Coriobacteriia | VIT D | -0.51 | 0.37 | 0.174 | -2.11 | 0.51 | <0.001 |
|  | PRO+VIT D | -2.61 | 0.36 | <0.001 |  |  |  |
| Erysipelotrichia | VIT D | -1.01 | 0.34 | 0.003 | -0.96 | 0.47 | 0.044 |
|  | PRO+VIT D | -1.97 | 0.33 | <0.001 |  |  |  |
| Negativicutes | VIT D | -0.25 | 0.75 | 0.738 | 2.23 | 1.04 | 0.032 |
|  | PRO+VIT D | 1.98 | 0.72 | 0.006 |  |  |  |

**Table 6. Total abundance of bacteria phylum before and after 4 weeks probiotics supplementation**

| Outcome | Intervention | Est | SE | P | Est_pairwise_ | SE_pairwise_ | P_pairwise_ |
| --- | --- | --- | --- | --- | --- | --- | --- |
| Firmicutues | VIT D | -0.75 | 0.32 | 0.021 | -0.93 | 0.45 | 0.038 |
|  | PRO+VIT D | -1.68 | 0.31 | <0.001 |  |  |  |
